# Analysis of Chemical Structure and Antibiofilm Properties of Exopolysaccharides from *Lactiplantibacillus plantarum* EIR/IF-1 Postbiotics

**DOI:** 10.3390/microorganisms10112200

**Published:** 2022-11-07

**Authors:** Basar Karaca, Ozan Haliscelik, Mervi Gursoy, Fadime Kiran, Vuokko Loimaranta, Eva Söderling, Ulvi Kahraman Gursoy

**Affiliations:** 1 Department of Periodontology, Institute of Dentistry, University of Turku, 20520 Turku, Finland; 2 Department of Biology, Faculty of Science, Ankara University, 06100 Ankara, Turkey; 3 Pharmabiotic Technologies Research Laboratory, Department of Biology, Faculty of Science, Ankara University, 06100 Ankara, Turkey; 4 Oral Health Care, Welfare Division, City of Turku, 20521 Turku, Finland

**Keywords:** antibiofilm, *Filifactor alocis*, *Fusobacterium nucleatum*, lactic acid bacteria, *Porphyromonas gingivalis*, postbiotics, *Prevotella denticola*

## Abstract

Previous studies have indicated that the exopolysaccharides of lactic acid bacteria exhibit antibiofilm activity against non-oral bacteria by preventing their initial adhesion to surfaces and by downregulating the expression of genes responsible for their biofilm formation. The aims of this study were to (1) characterize the exopolysaccharides (EPSs) of *Lactobacillus plantarum* EIR/IF-1 postbiotics, (2) test their antibiofilm effect on dual biofilms, and (3) evaluate their bacterial auto-aggregation, co-aggregation, and hydrocarbon-binding inhibitory activity. The EPSs were characterized by FTIR, HPLC, and thermogravimetric analysis. Bacterial auto- and co-aggregation were tested by Kolenbrander’s method and hydrocarbon binding was tested by Rosenberg’s method. Dual biofilms were formed by culturing *Fusobacterium nucleatum* ATCC 25586 with one of the following bacteria: *Prevotella denticola* ATCC 33185, *P. denticola* AHN 33266, *Porphyromonas gingivalis* ATCC 33277, *P. gingivalis* AHN 24155, and *Filifactor alocis* ATCC 35896. The EPSs contained fractions with different molecular weights (51 and 841 kDa) and monosaccharides of glucose, galactose, and fructose. The EPSs showed antibiofilm activity in all the biofilm models tested. The EPSs may have inhibited bacterial aggregation and binding to hydrocarbons by reducing bacterial hydrophobicity. In conclusion, the EPSs of *L. plantarum* EIR/IF-1, which consists of two major fractions, exhibited antibiofilm activity against oral bacteria, which can be explained by the inhibitory effect of EPSs on the auto-aggregation and co-aggregation of bacteria and their binding to hydrocarbons.

## 1. Introduction

The interactions of a variety of microorganisms under the influence of environmental changes result in an extremely dynamic ecosystem in the oral cavity. While the presence of a symbiotic relationship between commensal microorganisms and the host suppresses the excessive growth of pathogenic microorganisms, dysbiosis can alter the oral microbiota in favor of pathogens, ultimately leading to two common infectious diseases of the oral cavity, namely caries and periodontitis [1]. The basis of treatment for these diseases is mechanical and chemical removal of pathogenic biofilms to maintain a healthy symbiotic relationship between the host and the oral microbiota [2]. Other preventive approaches, such as the use of probiotics, have also been discussed. Although probiotics may have beneficial effects on intestinal diseases, there is no satisfactory evidence yet that similar beneficial properties can be achieved against the pathogenic oral microbiota [3]. In addition, the use of probiotics for oral health may pose a risk to immunocompromised individuals because probiotics are living cells. In addition, a decrease in pH may lead to a demineralization of tooth enamel due to the ability of probiotic bacteria to ferment various sugars [4,5].

Postbiotics are metabolic byproducts released by living bacteria or by bacterial lysis that can have beneficial effects on the host [6]. Postbiotics consist of metabolites of live probiotic bacteria such as organic acids, released exopolysaccharides, short-chain fatty acids, amino acids, flavonoids, terpenoids, and phenolic compounds. They are also composed of inactivated or dead cells or their lysed parts, such as cell surface proteins, peptidoglycan, cell-bound polysaccharides, and teichoic acids [7,8]. The use of postbiotics as cellular components or metabolites has several advantages, including a better stability and concentration-dependent formulation compared to probiotics [9].

The use of postbiotics in oral diseases, especially periodontitis, has been studied in recent years [10]. Most of these studies have focused on the antimicrobial and antibiofilm effects of postbiotics on oral pathogens. The postbiotics of *Lactobacillus fermentum*, *Lactobacillus salivarius*, and *Lactococcus lactis* can suppress the growth of opportunistic or pathogenic bacteria such as *Streptococcus sanguinis*, *Streptococcus mutans*, *Porphyromonas gingivalis*, *Fusobacterium nucleatum*, *Tannerella forsythia*, and *Treponema denticola* [11,12]. Cell-free supernatants of *Lactobacillus rhamnosus* and *Lactobacillus acidophilus* can exert an antibiofilm effect by impairing biofilm integrity and reducing the expression of the biofilm-associated genes of periodontal pathogens [13,14].

The probiotic strains of lactic acid bacteria (LAB) are generally recognized as safe (GRAS), and their metabolites, such as antimicrobial peptides, bacteriocins, aromatic compounds, organic acids, fatty acids, and exopolysaccharides (EPSs), fall within the scope of postbiotics [15]. Several LAB species, particularly those of the genus *Lactobacillus*, are capable of producing EPSs, and some of the EPSs identified to date by LAB have been shown to have biological functions, such as free radical scavenging, free cholesterol binding, gut microbiota modulation, antitumor, antimicrobial, and antibiofilm properties [8,15,16]. Previous studies have shown that the EPSs of LAB can have antibiofilm effects by altering the cell surfaces of bacteria, preventing initial adhesion to surfaces, and downregulating the expression of biofilm-associated genes [17]. EPSs isolated from *L. plantarum* can also reduce biofilm production by inhibiting indole production associated with quorum sensing, as shown in *Escherichia coli* [18]. Shetty et al. [19] also found that different EPS contents of *L. plantarum* can alter the outer membranes of multidrug-resistant bacteria, resulting in a higher antibiotic susceptibility in biofilms. However, there is no evidence in the literature to support the efficacy of *L. plantarum* EPSs, and particularly its antibiofilm activity, against the oral opportunists or pathogens investigated in this study.

In the present study, we hypothesized that the EPSs of the *L. plantarum* EIR/IF-1 postbiotics inhibit oral biofilm formation. Therefore, the objectives of this study were (1) to characterize the EPSs of *L. plantarum* EIR/IF-1 obtained from postbiotics, (2) to test the antibiofilm effect of EPSs on dual biofilms (a Gram-negative pathogenic biofilm model (*F. nucleatum* + *P. gingivalis*), a Gram-negative commensal/opportunistic biofilm model (*F. nucleatum* + *P. denticola*), and a Gram-positive pathogenic model (*F. nucleatum* + *Filifactor alocis*)), and (3) to evaluate the inhibitory effect of the EPSs on the auto-aggregation, co-aggregation, and hydrocarbon binding of bacteria (*F. nucleatum*, *P. gingivalis*, *P. denticola,* and *Fil. alocis*). The test bacteria were selected based on their contribution to periodontal health and periodontitis. *F. nucleatum* colonizes the healthy oral cavity, but also supports pathogenic biofilms that contribute to periodontal disease [20]. *P. gingivalis*, a Gram-negative oral anaerobe, is an important etiologic agent associated with the progression of periodontitis [21]. The genus *Prevotella*, including *P. denticola*, acts on the early and middle steps of oral biofilm formation with cellular adhesion for later colonizers and, in particular, supports the biofilm formation of *Aggregatibacter actinomycetemcomitans* [22,23]. Finally, *Fil. alocis*, a fastidious Gram-positive model, has recently been introduced as a diagnostic indicator of periodontal disease. Due to its ability to interact and exhibit virulence with various oral bacteria, *Fil. alocis* has become an important species for periodontal disease progression [24].

## 2. Materials and Methods

### 2.1. Culture Preparation of the L. plantarum EIR/IF-1 Strain for Obtaining Postbiotics

The strain *L. plantarum* EIR/IF-1 (NCBI GenBank Accession Number: MW057714.1) (EIR; the code of our culture collection, IF; infant feces, 1; the first isolate from the sample) was kindly provided by the Pharmabiotic Technologies Research Laboratory, Department of Biology, Faculty of Science, Ankara University. The strain *L. plantarum* EIR/IF-1 stored at −80 °C in 50% glycerol was cultured for 24 h at 37 °C on De Man, Rogosa, and Sharpe Agar (MRS) (Merck, Darmstadt, Germany) before postbiotics preparation. A typical colony of the strain was collected with a sterile loop and transferred to 20 mL of MRS broth (Merck, Darmstadt, Germany). This suspension was incubated for 18 h at 37 °C. Subsequently, this culture (≈10^8^ CFU/mL) was used to inoculate 1 L of MRS broth at an inoculation ratio of 2% (*v*/*v*). After a final incubation of 18 h at 37 °C (late log phase), the entire culture was centrifuged at 15,000× *g* for 20 min at room temperature. The culture supernatant (spent culture medium) and pellet were separated, and the supernatant was filtered through a membrane with a pore size of 0.22 μm (Sartorius, Göttingen, Germany) [25]. The filtered samples were then freeze-dried and powdered (freezing conditions of −20 °C, a vacuum pressure of 0.120 mB, and a condenser temperature of −58 °C; Martin Christ Gefriertrocknungsanlagen GmbH, Harz, Germany). One liter of culture supernatant yielded approximately 10 g of powder. The powdered samples were suspended in both sterile dH_2_O and Todd-Hewitt broth at a final concentration of 250 mg/mL. The suspensions were stored at −20 °C for future studies.

### 2.2. Extraction of EPSs from Postbiotics of L. plantarum EIR/IF-1 Strain

A volume of 200 mL of the previously obtained postbiotics (spent culture medium) was used to extract the released EPSs. Trichloroacetic acid (TCA; Merck, Darmstadt, Germany) was added to the filtered spent culture medium at a final concentration of 20% (chemical deproteinization) and incubated for 2 h at 4 °C. After incubation, samples were centrifuged at 4 °C for 20 min. A double volume of 95% ethanol (Merck, Germany) was added to the supernatant, and incubated overnight at 4 °C. The samples were then centrifuged at 6000 rpm for 30 min at 4 °C. The pellets were dried for 4 h at 37 °C. Finally, the dried EPSs were dissolved in 2 mL of distilled water [26]. In addition, the protein content of the EPS sample was determined by the Bradford assay using bovine serum albumin as a reference according to the manufacturer’s instructions (Bio-Rad Laboratories Inc., Hercules, CA, USA). A small amount of protein was detected in the extracted EPS (20 µg/mL).

### 2.3. Determination of Total Carbohydrates

The phenol–sulfuric acid method was used to determine EPS concentrations [27]. A total of 500 µL of phenol and 5 mL of sulfuric acid were added to 500 µL of EPSs suspension and incubated for 10 min at room temperature and for 20 min at 30 °C. The absorbance of the samples was measured using a microplate reader set to 490 nm. Concentration values were calculated based on the results of glucose standards prepared at different concentrations. Finally, a 5 mg/mL of stock EPSs solution was prepared in Todd-Hewitt broth, the culture medium used in further studies.

### 2.4. Characterization of EPSs

#### 2.4.1. Monosaccharide Composition Analysis

The extracted EPSs were hydrolyzed with 1 M sulfuric acid at 100 °C for 2 h and then neutralized with 1 N NaOH. The monosaccharide composition was analyzed by HPLC (Shimadzu, Prominence 20A Series, Kyoto, Japan) using a Shodex Sugar SP0810 250 × 4.6 mm column (Shodex, Lexington, NY, USA) with a refractive index detector. Deionized water was used as the mobile phase with a flow rate of 0.6 mL/min for 20 min and an injection volume of 20 μL. The temperature of the column oven was set at 80 °C. Quantitative measurement was performed by establishing a linear calibration curve using the derivatives of standard sugars (fructose, glucose, sucrose, galactose, xylose, arabinose, and mannose). All unknown samples were diluted with deionized water and filtered using a nylon filter (Merck, Darmstadt, Germany). Sugar composition was detected at HPLC-RID and measured against the calibration curve of the free sugar standard mixture.

#### 2.4.2. Fourier Transform Infrared Spectroscopy Analysis

The functional chemical groups of the extracted EPSs were analyzed by Fourier transform infrared spectroscopy (FTIR) using the attenuated total reflectance (ATR) method (IR Spirit Infrared Spectrometer, Shimadzu, Kyoto, Japan). Spectra were recorded between 4000 and 600 cm^−1^, with a spectral resolution of 2 cm^−1^. The absorption of the background and samples was measured with 100 scans.

#### 2.4.3. Determination of Molecular Weight

The molecular weight of the EPSs was determined by gel permeation/size exclusion chromatography (Shimadzu-Prominence 20A series, Kyoto, Japan) using a PSS SUPREMA 5 µm GPC column 250 × 10 mm with a refractive index detector. The EPSs sample and all dextran standard samples were weighed to 10 mg and dissolved in 3 mL of distilled water. Dextran standards ranging from 20,000 to 1,000,000 Da were used for the parabolic standard calibration curve in the GPC software. Deionized water was used as the mobile phase with a flow rate of 1 mL/min for 30 min and an injection volume of 20 μL.

#### 2.4.4. Thermogravimetric Analysis (TGA) of EPSs

Thermogravimetric analysis of EPSs was performed with Shimadzu TGA 50 and Diamond TGA instruments using 10 mg of EPSs. The TGA curve represents the temperature of the reference material on the -axis against the TGA signal, which was converted into a percent change in weight on the *x*-axis. The EPSs were placed in a platinum crucible and heated at a linear rate of 20 °C/min over a temperature range of 40 °C–720 °C for 34 min under nitrogen, and the corresponding weight loss was determined.

### 2.5. Test Bacteria and Culture Conditions

*P. denticola* ATCC 33185 (type strain), *P. denticola* AHN 32366 (clinical strain), *P. gingivalis* ATCC 33277 (type strain), *P. gingivalis* AHN 24155 (clinical strain), *F. nucleatum* ATCC 25586 (type strain), and *Fil. alocis* ATCC 35896 (type strain) were provided by the culture collections of the Institute of Dentistry, University of Turku, and preferred as test bacteria in all experiments.

All strains stored at −70 °C in milk were cultured on Brucella blood agar medium supplemented with 750 mg/mL cysteine, 5 mg/mL hemin, and 10 mg/mL vitamin K1. All strains were incubated under anaerobic conditions (10% H_2_, 5% CO_2_, and 85% N_2_, Whitley A35 Anaerobic Workstation, Don Whitley Scientific Ltd., Bingley, UK) at 37 °C for 5 days prior to experiments.

### 2.6. Antibiofilm Activity of EPSs

Considering interspecies interactions in oral biofilms, the following dual biofilm models with *F. nucleatum* ATCC 25586 were developed to determine the antibiofilm effects of EPSs [28,29]:

*P. denticola* ATCC 33185 + *F. nucleatum* ATCC 25586;

*P. denticola* AHN 32366 + *F. nucleatum* ATCC 25586;

*P. gingivalis* ATCC 33277 + *F. nucleatum* ATCC 25586;

*P. gingivalis* AHN 24155 + *F. nucleatum* ATCC 25586;

*Fil. Alocis* ATCC 35896 + *F. nucleatum* ATCC 25586.

Activated cultures on Brucella blood agar were used for all biofilm tests. Typical colonies of each strain were harvested from the plates and suspended in sterile physiological serum (0.9% NaCl) to obtain an optical density (OD_490nm_) value of 2.0 for strains of *P. denticola*, *F. nucleatum*, and *Fil. alocis* and a value of 2.5 OD for the strains of *P. gingivalis* (≈10^9^ CFU/mL for each strain) (Shimadzu UV-visible, BioSpec-mini, Kyoto, Japan). To prepare different combinations as described above, equal volumes (7.5 µL) of each bacterial suspension were mixed (15 µL in total).

Antibiofilm experiments were performed using three different approaches: co-treatment, pre-treatment, and post-treatment.

#### 2.6.1. Co-Treatment Assay

In order to test the effects of EPSs during biofilm formation, the use of co-treatment was preferred. For all biofilm experiments, 96-well polystyrene microtiter plates were used. The plates were coated with pasteurized human saliva as described by Fteita et al. [30]. Briefly, 10 mL of previously collected saliva from 4 systemically healthy, non-smoking volunteers was pooled and centrifuged at 10,000 rpm and 4 °C for 40 min. The salivary pellet was removed from the supernatant, and the supernatant was pasteurized at 60 °C for 30 min. The pasteurized saliva was then centrifuged again and clarified.

Each well of the plates was filled with 100 µL of pasteurized saliva and the plates were incubated at 37 °C for 1 h. The plates were then emptied and 135 µL of Todd-Hewitt broth (casein peptone 10 g/L, heart infusion 3.1 g/L, sodium carbonate 2.5 g/L, dextrose 2 g/L, sodium chloride 2 g/L, and disodium phosphate 0.4 g/L; supplemented with 750 mg/mL cysteine, 5 mg/mL hemin, and 10 mg/mL vitamin K1) adjusted with various concentrations of EPSs (25, 50, 125, 250, and 500 µg/mL) was added. Mixed bacterial suspensions (15 µL) were added to each well and the plates were incubated at 37 °C for 48 h under anaerobic conditions. Wells containing inoculum and medium were considered positive controls, while wells containing only medium with different concentrations of EPSs were considered negative controls. After incubation, the amount of accumulated biofilm was measured using the crystal-violet binding assay. Briefly, after incubation, the plates were emptied and washed three times with saline. Each well was filled with 95% methanol and incubated for 10 min at room temperature. Then, the wells were emptied and filled with 0.1% crystal-violet solution and the plates were incubated at room temperature for 30 min. Finally, the plates were rinsed with distilled water and 33% acetic acid solution was added to each well and incubated for 15 min. The OD values were measured using an ELISA reader at a wavelength of 595 nm (Multiskan FC Microplate Photometer, Thermo Fisher Scientific, Waltham, MA, USA) [31].

#### 2.6.2. Pre-Treatment Assay

This approach was used to test how biofilm formation is affected when saliva-coated microplate wells are treated with EPSs prior to biofilm sampling. At this stage, culture preparations and coating of the plates with saliva were first performed as previously described. After coating with saliva, the wells were emptied and 150 µL of Todd-Hewitt broth medium containing EPSs (25, 50, 125, 250 and 500 µg/mL) was added to each test well. Only the medium was added to the wells of the positive and negative controls. The plates were incubated at 37 °C for 24 h. At the end of incubation, the plates were emptied and 135 µL of Todd-Hewitt broth medium was added to the wells. With the exception of the negative control wells, 15 µL of the mixed suspensions was added to other wells. The plates were incubated for 48 h as previously described and subjected to the crystal-violet binding assay.

#### 2.6.3. Post-Treatment Assay

This application tested the eradicating effect of EPSs on established biofilms. First, the plates were coated with saliva and 135 µL of Todd-Hewitt broth was added to each well and inoculated with 15 µL of the prepared mixed culture suspensions (except for the negative control wells, which received medium only). The plates were incubated for 48 h as described above. At the end of incubation, the wells were emptied and carefully washed twice with phosphate-buffered saline (PBS, pH 7.2) and filled with 150 µL of Todd-Hewitt broth containing various concentrations of EPSs (25, 50, 125, 250, and 500 µg/mL). Both positive and negative control wells were filled with media only. Plates were incubated anaerobically for 24 h, rinsed, and subjected to the crystal-violet binding assay.

### 2.7. Testing EPSs on Auto-Aggregation and Co-Aggregation

EPSs were used to test their anti-aggregation capabilities. The aggregation tests were performed to understand the potential antibiofilm mode of action of EPSs. The aggregation tests were performed according to Kolenbrander’s method with modifications [32]. Test strains were cultured on Brucella blood agar plates under previously described conditions. A few colonies were collected from each plate with a sterile loop and transferred to 10 mL of Todd-Hewitt broth. The culture suspensions were incubated overnight under anaerobic conditions and centrifuged at 12,000× *g* for 10 min. After removal of the supernatant, the cells were washed once with PBS. Another centrifugation at 12,000× *g* for 10 min took place, and the cells were resuspended in the co-aggregation buffer (Tris HCl, pH 8.0, containing 0.1 mM CaCl_2_, 0.1 mM MgCl_2_, and 150 mM NaCl, pH 8.0). The suspensions of *P. denticola*, *F. nucleatum*, and *Fil. alocis* were optically adjusted to 0.7 OD at 660 nm (Shimadzu UV-visible, BioSpec-mini, Kyoto, Japan). These cultures corresponded to approximately 10^8^ CFU/mL (validated by colony count on Brucella blood agar). *P. gingivalis* strains were also adjusted to ≈10^8^ CFU/mL (optical density of 1.0 at 660 nm).

For the auto-aggregation experiments, 1 mL culture suspensions of each strain without EPSs were prepared as control groups and 1 mL culture suspensions with different concentrations of EPSs (100, 250, and 500 µg/mL) were prepared as test groups. The preferred concentrations of EPSs had no antibacterial effect (which was verified before the aggregation test). For the co-aggregation experiments, *F. nuclaetum* ATCC 25586 was preferred due to its high aggregation ability. A total of 500 µL of each suspension was mixed with 500 µL of the *F. nucleatum* ATCC 25586 suspension and EPSs were added. The final concentrations of the suspensions were adjusted for EPSs by addition to the final suspensions. All suspensions were vigorously shaken with a vortex for 2 min before optical measurement at 660 nm and measured immediately (t_0_). The measurements of the suspensions were performed at different time intervals (t_1_: 1 h, t_2_: 2 h, t_3_: 3 h, t_24_: 24 h). During the time intervals, the test cuvettes were covered with paraffin strips at 37 °C. The optical measurements were recorded.

### 2.8. Adhesion to Hydrocarbons

The adhesion of hydrocarbons to test strains was evaluated to determine the hydrophobicity of the cell surface. This experiment was also preferred to understand the potential antibiofilm mode of action of EPSs. Adhesion to hydrocarbons was performed using a modified version of Rosenberg’s method [33]. Liquid hydrocarbons, such as toluene and xylene, were used to evaluate the degree of adhesion of bacterial cells under the influence of EPSs.

Bacterial suspensions were prepared according to the steps described in the previous Section, except that the final culture suspensions were prepared with PBS solution (pH 7.2). Different concentrations of EPSs (250 and 500 µg/mL) were added to 1.2 mL of each bacterial suspension. Suspensions without EPSs served as control groups. These suspensions were shaken for 2 min at maximum intensity using a vortex mixer. Prior to the addition of the hydrocarbons, the optical density of the suspensions was measured at a wavelength of 660 nm (t_0_). The suspensions were transferred into 10 mL glass tubes and 0.5 mL of toluene or xylene was added to each tube. The tubes were shaken with a vortex for 2 min and allowed to stand at room temperature for 30 min. Finally, 1 mL of each suspension was taken from the lower phase and transferred into plastic cuvettes and measured at OD_660nm_. The formula for calculating the percent hydrophobicity of the cell surface was 100 × [(initial OD_660nm_ − final OD_660nm_)/(initial OD_660nm_)].

### 2.9. Statistical Analysis

Antibiofilm experiments were performed in biological triplicates and six technical replicates in each experiment. Aggregation and hydrocarbon-binding tests were performed in biological and technical triplicates. The mean and standard deviation of the data were given. One-way ANOVA test was used for comparisons between the groups and Tukey’s test for post hoc analyses in antibiofilm experiments. Two-way ANOVA was used in an auto- or co-aggregation assay to examine how the means change with time and EPSs concentrations. *p*-values of <0.05, <0.01, <0.001, and <0.0001 were accepted as statistically significant. Prism GraphPad 8.0.1 (Graph Software Inc, San Diego, CA, USA) was used for the statistical analysis and the generation of the figures.

## 3. Results

### 3.1. Characterization of EPSs

#### 3.1.1. Monosaccharide Composition Analysis

**Figure 1:**
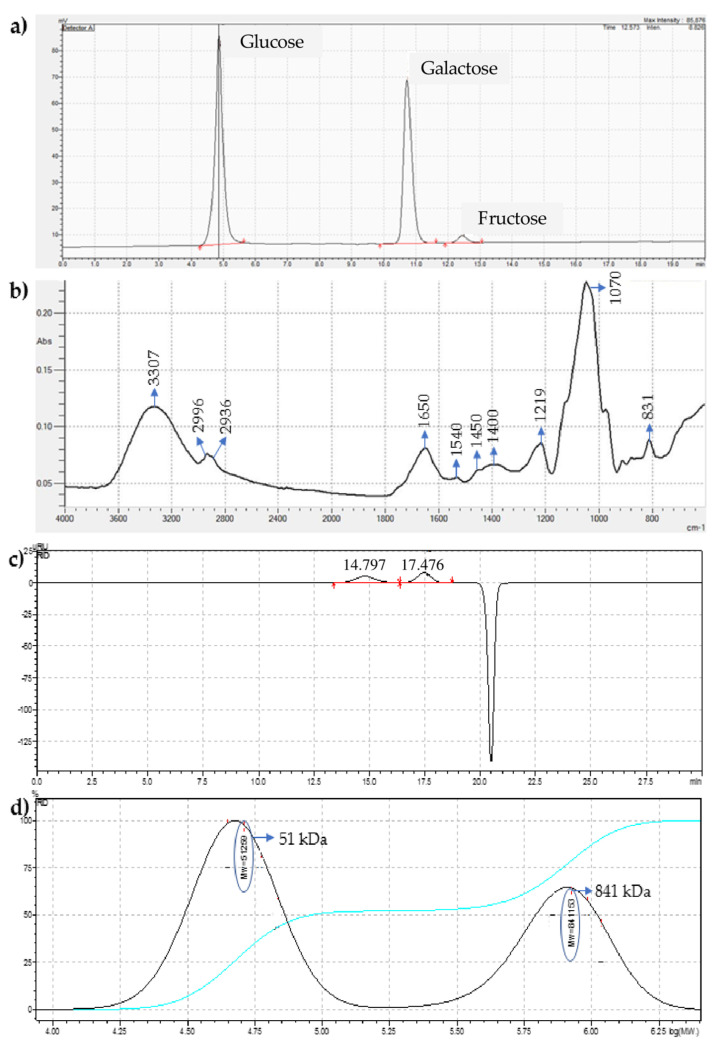
(**a**) Hydrolyzed exopolysaccharides (EPSs) from *L. plantarum* EIR/IF-1, (**b**) FTIR spectrum of EPSs, (**c**) Gel permeation chromatogram of EPSs, (**d**) molar mass distribution (MMD) pattern. Molecular weight of two fractions in EPSs are indicated in blue circles.

The results were evaluated with respect to the mono- and disaccharides in EPSs. The HPLC chromatogram showed that glucose was the highest saccharide with a concentration of 5.674 g/100 g. Galactose and fructose were found in EPSs at 4.476 g/100 g and 0.23 g/100 g, respectively. Overall, all the saccharides were detected in statistically significant amounts in the EPSs (Figure 1a). The presence of various monosaccharides indicates that the EPS is a heteropolysaccharide.

#### 3.1.2. Fourier Transform Infrared Spectroscopy Analysis

An FTIR spectrum was recorded in absorption mode from 4000 to 400 cm^−1^ to investigate the functional groups of EPSs (Figure 1b). Polysaccharides have a large number of hydroxyl groups that exhibit a strong broad stretching peak at 3307 cm^−1^. Due to the hydrogen bonds formed between the different hydroxyl groups, the absorption in this region exhibits a round shape, which is typical of most O-H stretching modes, indicating that the molecule is an EPS [34]. Two weak C-H stretching peaks at 2966 and 2936 cm^−1^ in the FTIR spectra of *L. plantarum* EIR/IF-1 EPSs show both methyl groups and methylene groups, respectively [35]. A strong absorption at 1652 cm^−1^ corresponds to the amide I > C=O stretching and the C-N bending of protein and peptide amines [35,36]. Another weak peak at 1540 cm^−1^ can be considered as the N-H bending of the amides II of the proteins [37]. The peak at 1450 cm^−1^ was assigned to the C-H bending in the CH3 groups or aromatic -C=C stretching vibrations in proteins [36]. A medium peak at 1400 cm^−1^ was considered as a >C=O stretching of the COO- groups and a C-O bending of the COO- groups [36]. The peak at 1219 cm^−1^ could be assigned to the C-O stretching in the ether or alcohol groups [38]. A strong and broad peak at 1000–1200 cm^−1^ indicates that the analyzed molecule is a carbohydrate, since C-O-C and C-O are present [39]. FTIR spectroscopy can characterize the sample as a whole molecule below the 1500 cm^−1^ region and the peak at 1070 cm^−1^ indicates that the molecule is a polysaccharide. The peak at 836 cm^−1^ is characteristic of α-D-glucan [40] (Figure 1b).

#### 3.1.3. Molecular Weight Estimation of EPSs

In size exclusion chromatography or gel permeation chromatography, the analytes are separated based on their molecular size. A calibration curve was constructed using the logarithm of molecular weight as a function of retention time with the dextran standards. The chromatogram of the EPSs appeared as two peaks at 14.8 and 17.5 min, confirming the heterogeneity of the extracted EPSs sample (Figure 1c). Two fractions with different molecular weights were obtained: 51 kDa and 841 kDa (Figure 1d). The negative peak at 20.6 min was a system peak in all the measurements. This peak was obtained in all the chromatograms due to the relationship between eluent and the column. The peak at 20.6 min did not affect the measurements.

#### 3.1.4. Thermogravimetric Analysis (TGA) of EPSs

A thermogravimetric analysis of the EPSs was dynamically performed between temperature and weight loss. When the EPSs are exposed to different temperatures, the first temperature increase primarily leads to gelatinization and swelling. A further increase in temperature leads to the dehydration and pyrolysis of the exopolysaccharide.

A two-stage weight loss due to dehydration was observed during the thermogravimetric analysis of the EPSs (Figure S1, Supplementary File). The EPSs showed an initial weight loss between 50 °C and 130 °C. This initial weight loss could be due to the loss of moisture by the carboxyl groups, which are present in large amounts and bound to water molecules. Thus, the initial weight loss of the EPSs was due to the presence of a high content of carboxyl groups. The second weight loss was observed at a degradation temperature of 129.20 °C.

### 3.2. Antibiofilm Activity of EPSs

**Figure 2:**
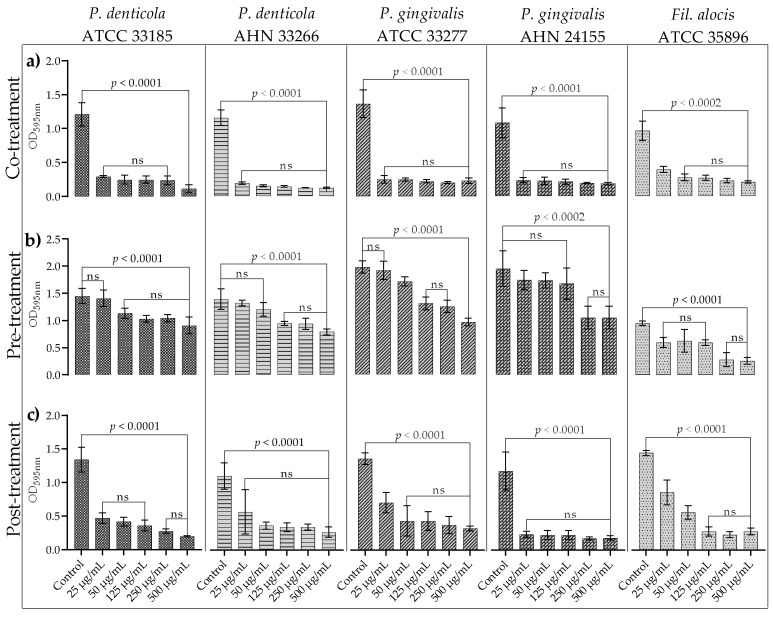
Antibiofilm activity of EPSs. All biofilms are dual-species biofilms including *F. nucleatum* ATCC 25586. Co-treatment assay: Biofilm formation in culture media adjusted with different concentrations of EPSs. (**a**) *P. denticola* ATCC 33185, *P. denticola* AHN 33266, *P. gingivalis* ATCC 33277, *P. gingivalis* AHN 24155, *Fil. alocis* ATCC 35896, respectively, in row (**a**). Pre-treatment assay: Biofilm formation on treated surfaces with different concentrations of EPSs (**b**) *P. denticola* ATCC 33185, *P. denticola* AHN 33266, *P. gingivalis* ATCC 33277, *P. gingivalis* AHN 24155, *Fil. Alocis* ATCC 35896, respectively, in row (**b**). Post-treatment assay: EPSs treatment with different concentrations on established biofilms. (**c**) *P. denticola* ATCC 33185, *P. denticola* AHN 33266, *P. gingivalis* ATCC 33277, *P. gingivalis* AHN 24155, *Fil. alocis* ATCC 35896, respectively, in row (**c**). ns: not significant. Bars include standard deviation. *Y*-axis was equalized in each row.

In order to determine whether the extracted EPSs affected the biofilms of the test bacteria, co-treatment, pre-treatment, and post-treatment experiments were conducted. As shown in the co-treatment experiments (Figure 2a), all the concentrations of EPSs proved sufficient to inhibit biofilm formation (*p* < 0.0001 for *P. d* ATCC 33185 + *F. n* ATCC 25586, *P. d* AHN 33266 + *F. n* ATCC 25586, *P. g* ATCC 33277 + *F. n* ATCC 25586, *P. g* AHN 24155 + *F. n* ATCC 25586, and *p* < 0.0002 for *F. a* ATCC 35896 + *F. n* ATCC 25586). Although the tested EPS concentrations did not exhibit antimicrobial activity, a very effective inhibition of biofilm formation was observed for all test strains. The testing of the EPSs on the growth of the test strains showed no antimicrobial activity. The test strains were cultured in 96-well polystyrene plates for 48 h at 37 °C under anaerobic conditions with the indicated EPS concentrations in biofilm experiments (Supplementary File, Figure S2).

A decrease in biofilm production was observed in all strains, including *P. d* ATCC 33185 + *F. n* ATCC 25586, due to the increasing EPS concentrations (*p* < 0.0001). While EPSs at a concentration of 25 µg/mL were found to be ineffective in *P. d* ATCC 33185 + *F. n* ATCC 25586, the extent of biofilm production in *P. d* AHN 33266 + *F. n* ATCC 25586 under the influence of 25 and 50 µg/mL concentrations did not differ from the control group. Moreover, higher EPS concentrations were very effective for the pre-treatment of these biofilms (*p* < 0.0001). For *P. g* ATCC 33277 + *F. n* ATCC 25586, only the 25 µg/mL EPSs concentration proved ineffective, while for *P. g* AHN 24155 + *F. n* ATCC 25586, a significant decrease in biofilm production was observed only at the 250 and 500 µg/mL EPS concentrations (*p* < 0.0001 and *p* < 0.0002, respectively). For *F. a* ATCC 35896 + *F. n* ATCC 25586, a significant decrease in biofilm formation was observed at all EPS concentrations in an increasing trend (*p* < 0.0001) (Figure 2b).

All tested concentrations of the EPSs were effective in eradicating mature biofilms (Figure 2c) (*p* < 0.0001 for all groups).

### 3.3. Investigation of Antibiofilm Activity of EPSs in Terms of Hydrophobicity and Cell–Cell Interactions

#### 3.3.1. Testing EPSs on Auto-Aggregation and Co-Aggregation

**Figure 3:**
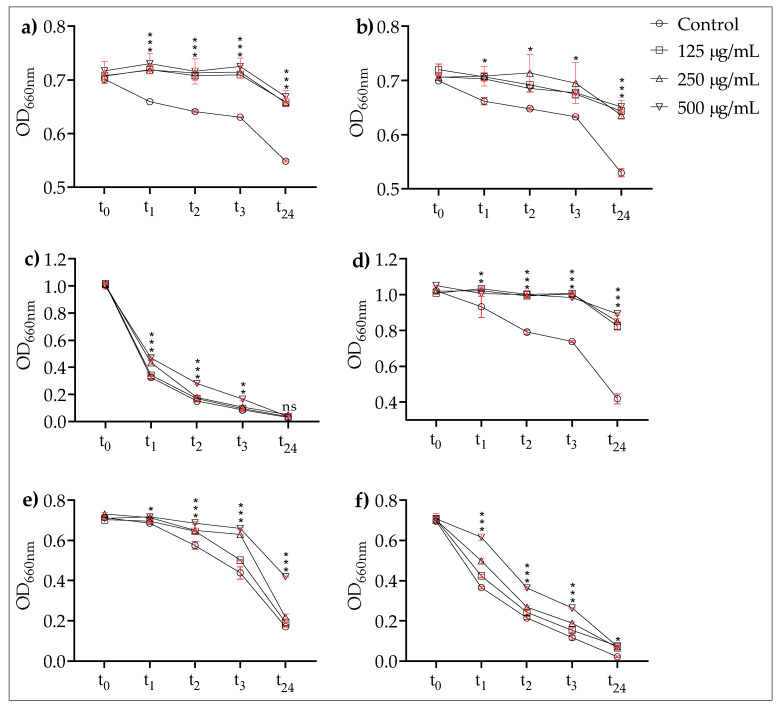
Effects of EPSs on auto-aggregation. (**a**) *P. denticola* ATCC. (**b**) *P. denticola* AHN 33266. (**c**) *P. gingivalis* ATCC 33277. (**d**) *P. gingivalis* AHN 24155. (**e**) *Fil. alocis* ATCC 35896. (**f**) *F. nucleatum* ATCC 25586. ns: not significant, ***: *p* < 0.001; **: *p* < 0.01; *: *p* < 0.05. Dots include standard deviation. *Y*-axis was equalized in each row.

**Figure 4:**
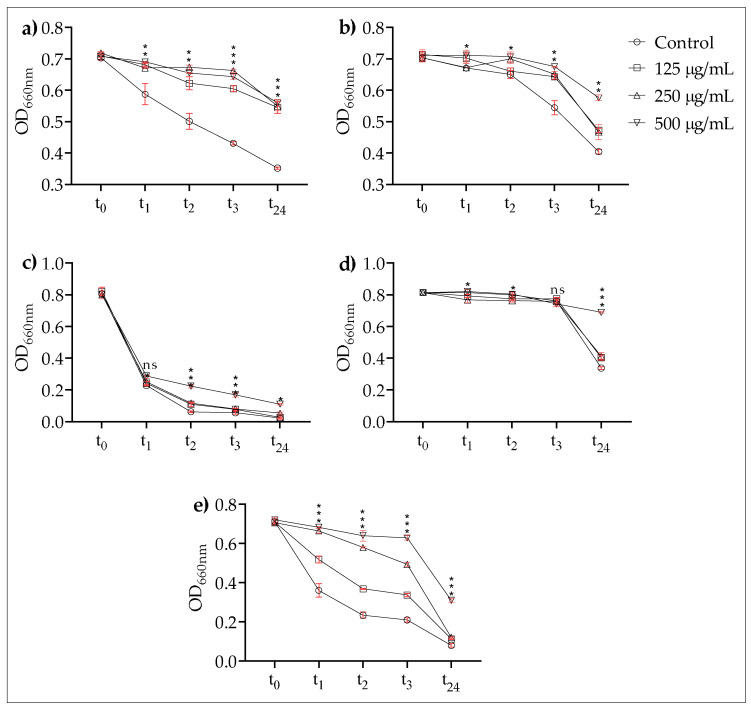
Effects of EPS on co-aggregation. (**a**) *P. denticola* ATCC. (**b**) *P. denticola* AHN 33266. (**c**) *P. gingivalis* ATCC 33277. (**d**) *P. gingivalis* AHN 24155. (**e**) *Fil. alocis* ATCC 35896. ns: not significant, ***: *p* < 0.001; **: *p* < 0.01; *: *p* < 0.05. Dots include standard deviation.

The EPSs had inhibitory effects both on the auto-aggregation and co-aggregation levels of all the tested strains. The auto-aggregation and co-aggregation results of the EPSs are given in Figure 3 and 4. The EPSs generally delayed auto-aggregation. At the end of the 24 h incubation period, the effects of the tested EPS concentrations on the auto-aggregation rates of *P. gingivalis* ATCC 33277 and *F. nucleatum* ATCC 25586 had almost disappeared, whereas the effects within the first three hours were partially preserved. Only at the highest EPSs concentration (500 µg/mL), a partial effect remained (Figure 3c,f). In *P. denticola* ATCC 33185, *P. denticola* AHN 33266, *P. gingivalis* AHN 24155, and *Fil. alocis* ATCC 35896, the EPSs significantly inhibited the auto-aggregation rates at all incubation times (Figure 3a,b,d,e).

The efficacy of EPSs on co-aggregation rates again showed a very similar trend. For all dual cultures, the change in optical density varied as a function of the different concentrations, i.e., higher concentrations were found to be more effective. Even after 24 h, the test groups of *P. denticola* strains had higher optical values than the control group at all the tested concentrations (Figure 4a,b). A rapid decrease in optical density of dual culture with *P. gingivalis* ATCC 33277 was observed, similar to the auto-aggregation rate, under the influence of EPSs (Figure 4c). While no remarkable results were found regarding the effects of EPSs on the co-aggregation rates in *P. gingivalis* AHN 24155, except for the highest concentration of 500 µg/mL, EPSs proved to be more effective in *Fil. alocis* ATCC 35896.

#### 3.3.2. Adhesion to Hydrocarbons

**Figure 5:**
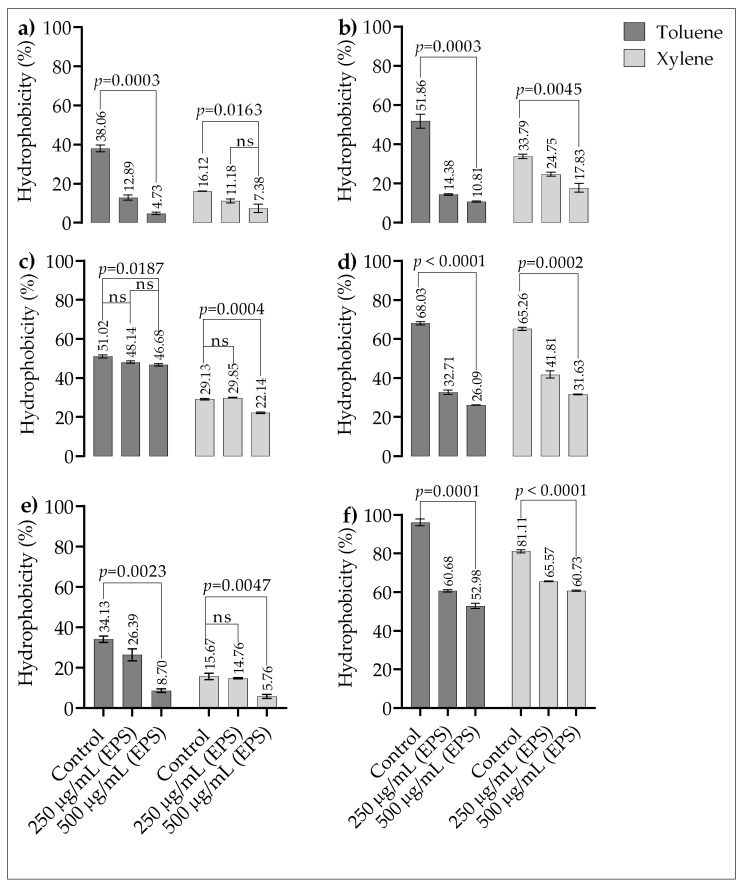
(**a**) *P. denticola* ATCC 33185. (**b**) *P. denticola* AHN 33266. (**c**) *P. gingivalis* ATCC 33277. (**d**) *P. gingivalis* AHN 24155. (**e**) *F. nucleatum* ATCC 25586. (**f**) *Fil. alocis* ATCC 35896. Bars include standard deviation. EPS: exopolysaccharide. ns: not significant. Bars include standard deviation.

In the strains *P. denticola* ATCC 33185 and *P. denticola* AHN 33266, binding affinity was found to decrease significantly for both toluene and xylene, which was attributed to the decrease in the hydrophobicity of the cell due to increasing EPS concentrations (Figure 5a,b). For the strain *P. gingivalis* ATCC 33277, no significant change in the binding of toluene was observed at any EPSs concentration, whereas for xylene, a decrease was observed only at an EPSs concentration of 500 µg/mL (Figure 5c). However, in contrast to the strain *P. gingivalis* ATCC 33277, a decrease in the binding affinity of toluene and xylene was observed for *P*. *gingivalis* AHN 24155 at all the tested EPS concentrations (Figure 5d). While 250 µg/mL EPSs did not alter the binding to xylene in *Fil. alocis* ATCC 35896, both EPS concentrations tested were effective in toluene (Figure 5e). Another remarkable result regarding EPS-induced decrease in cell hydrophobicity was found in the strain *F. nucleatum* ATCC 25586 (Figure 5f).

## 4. Discussion

To our knowledge, this is the first study to (1) demonstrate the antibiofilm effect of *L. plantarum* EIR/IF-1 EPSs from postbiotics on oral biofilms and to (2) explain the antibiofilm effect of EPSs through their strong inhibitory effects on bacterial auto-aggregation, co-aggregation, and hydrocarbon binding.

According to our findings, the EPSs obtained from the spent culture medium of the strain *L. plantarum* EIR/IF-1 contain two different fractions of 51 and 841 kDa. The small fraction may be derived from the viscous portion of the culture medium obtained from the centrifuged pellets, and this viscous portion may contain cell-bound capsular polysaccharides loosely adhered to the cell surface [41]. The extraction of EPSs into two fractions has been previously observed in lactic acid bacterial strains such as *L. lactis* subsp. *cremoris*, *Lactobacillus casei* subsp. *rhamnosus*, and *L. plantarum* [26,42,43]. With regard to the molecular weights of the EPS fractions obtained in the current study, we note that the results obtained by Tallon et al. [26] with the *L. plantarum* EP56 strain are very similar. While the weight of the cell-bound EPS in the aforementioned study was calculated to be about 850 kDa, the other EPS fraction, which is a smaller fraction released into the medium, was calculated to be about 40 kDa. The molecular weight of EPSs is an important factor that can affect their biological function [17]. In this study, it was found that the EPS fraction with a weight of 841 kDa could be the fraction responsible for the antibiofilm activity. Considering the data in the literature, it was found that antibiofilm activity is observed when a large EPS fraction is used [44].

According to our findings, the monosaccharide composition of the EPSs from *L. plantarum* EIR/IF-1 consists of glucose, galactose, and fructose. Various *L. plantarum* strains have already been shown to synthesize a variety of heteropolysaccharides. While the EPSs of *L. plantarum* YW32 consist of mannose, fructose, galactose, and glucose, *L. plantarum* E P56 is capable of producing an EPS consisting of glucose, galactose, and N-acetyl galactosamine. Another NTU 102 strain of *L. plantarum* can produce fructose, arabinose, galactose, glucose, mannose, and maltose in a different molar ratio [17,26,45]. The type and origin of the strains, the culture conditions, and the composition of the medium can influence the content of the monosaccharides in the EPSs produced by LAB [46]. Galactose and glucose are the most abundant and frequently determined monosaccharides in the heteropolysaccharides of the strains of *L. plantarum* of human origin [47]. Considering the glucose and galactose content (ratio 5.67:4.47) in the EPSs content of the strain *L. plantarum* EIR/IF-1 and the fact that this strain was isolated from infant feces, there is an overlap with the results of Salazar et al. [47].

The evaluation of the FTIR analysis of *L. plantarum* EIR/IF-1 EPS shows that no monosaccharide is substituted by phosphate, acetyl, or glyceryl in the obtained peaks. A medium peak around 1400 cm^−1^, a strong and broad peak at 1000–1200 cm^−1^, and the results of the TGA analysis showed that the EPS of *L. plantarum* EIR/IF-1 is an exopolysaccharide with functional carboxylate groups [35,36,39]. The strong absorption at 1652 cm^−1^ showed that the analyzed EPS is a heteropolysaccharide containing monosaccharide amine [36]. The monosaccharide amine, which we determined in the FTIR analysis, could not be detected with HPLC because it was not an appropriate standard for this study. In this study, the EPSs of *L. plantarum* EIR/IF-1 were characterized as far as possible, and an attempt was made to speculate on the mechanism of antibiofilm activity based on the chemical properties of the EPSs. However, the presence of possible additional functional groups such as phosphate or sulfonate and the determination of the total charge of the EPSs will be useful to better explain the mode of antibiofilm action.

In the present study, the antibiofilm capabilities of *L. plantarum* EIR/IF-1 EPSs were tested on a Gram-negative pathogenic biofilm model (*F. nucleatum* + *P. gingivalis*), a Gram-negative commensal/opportunistic biofilm model (*F. nucleatum* + *P. denticola*), and a Gram-positive pathogenic model (*F. nucleatum* + *Fil.* alocis). The antibiofilm properties of *L. plantarum* EIR/IF-1 EPSs were tested for the first time in the elimination of biofilms developed by *P. denticola* strains together with *F. nucleatum*, and effective results were obtained, especially when the co-treatment was also applied. For the *P. gingivalis*-*F. nucleatum* and *Fil. alocis*-*F. nucleatum* biofilms, pre-treatment and post-treatment applications were also found to be remarkably effective, with co-treatment being the most effective. These results suggest that *L. plantarum* EIR/IF-1 EPSs have different antibiofilm activities at the species and strain levels. Finally, this study clearly demonstrated that *L. plantarum* EIR/IF-1 EPSs were effective in all approaches to antibiofilm application. Moreover, it was noteworthy that the inhibition of biofilm formation or eradication of the tested strains was achieved in all antibiofilm applications of EPSs without strain differentiation. In conclusion, the pure form of EPS, which is a postbiotic component, is a stronger antibiofilm agent to understand the general mechanism of antibiofilm activity of postbiotics on the tested strains. This result also shows that the characterization of postbiotics, which are a complex cocktail, is crucial depending on the biological functions studied.

The EPSs of *L. plantarum* EIR/IF-1 significantly inhibited the auto- and co-aggregation abilities of the tested strains. It is very likely that this result is related to the anti-aggregative activity of the monosaccharide composition of *L. plantarum* EIR/IF-1 EPSs. The first step, which corresponds to the adhesion of early colonizers in the oral cavity, is crucial for biofilm formation. *F. nucleatum* acts as a bridge between a variety of colonizers by co-aggregation [20]. Co-aggregation within different oral species is also a crucial process for the build-up of complex biofilms. Thus, the in vitro co-aggregation assay can be a simple and appropriate model to examine oral biofilm formation [20]. It is a well-known phenomenon that some sugars such as lactose, D-fructose, D-fucose, and D-galactose can mask cell lectins and alter some cell surface properties [48]. *F. nucleatum* and *P. gingivalis* can bind carbohydrate components on cell surfaces, and *F. nucleatum,* in particular, has a galactose-binding lectin that recognizes the cell receptors of some bacteria, leading to co-aggregation [48,49,50]. It can therefore be assumed that the anti-aggregative properties of *L. plantarum* EIR/IF-1 EPSs can be related to their galactose residues.

As can be seen from our results, the EPSs reduced the hydrophobicity and adherence to hydrocarbons. Cell surface hydrophobicity is another key factor contributing to bacterial biofilm formation, including oral bacteria such as *P. gingivalis* and *F. nucleatum* [51,52]. Some EPSs derived from bacteria, including LAB, can act as surfactants by modifying the cell surface and reducing hydrophobicity [43]. In particular, negatively charged EPSs have a repulsive effect by hindering cell–cell interaction of negatively charged Gram-negative cell membranes. Considering the FTIR analysis of *L. plantarum* EIR/IF-1 EPSs, it is clear that EPS is a negatively charged molecule in terms of its functional groups, especially carboxylic groups. As such, this EPS may have acted as an anionic surfactant and reduced the binding affinity of the tested bacteria to hydrocarbons [53].

## 5. Conclusions

In the context of this in vitro study, it can be concluded that the EPSs of *L. plantarum* EIR/IF-1 from postbiotics, consisting of two fractions of 51 and 841 kDa, exhibit antibiofilm activity against oral bacteria, which can be explained by the inhibitory effect of EPSs on bacterial auto-aggregation, co-aggregation, and hydrocarbon binding. Furthermore, future studies may consider separating the EPSs of *L. plantarum* EIR/IF-1 with two different molecular weights into fractions to test each fraction individually. Finally, in addition to the antibiofilm activity of this EPS, by preventing bacterial aggregation and binding to hydrocarbons investigated in this study, the potential of EPS to suppress adhesin production or the quorum-sensing mechanism in target bacteria should also be investigated.

## Data Availability

The datasets generated during and/or analyzed during the current study are available from the corresponding author/s.

## Supplementary Materials

The following supporting information can be downloaded at: https://www.mdpi.com/article/10.3390/microorganisms10112200/s1, Figure S1 (TG curves of *L. plantarum* EIR/IF-1 EPS fraction) and Figure S2 (Testing of EPS fractions for growth of test strains. No antimicrobial activity was observed. Test strains were cultured in 96-well polystyrene plates for 48 h at 37 °C under anaerobic conditions with the indicated EPS concentrations in biofilm experiments).
