# Supplementary material for: Analysis of Chemical Structure and Antibiofilm Properties of Exopolysaccharides from Lactiplantibacillus plantarum EIR/IF-1 Postbiotics
Source: Microorganisms. 2022 Nov 7;10(11):2200. doi: 10.3390/microorganisms10112200 (PMC9693231; doi:10.3390/microorganisms10112200)
Supplement: Supplementary file 1 [file microorganisms-10-02200-s001.zip › microorganisms-1993445-supplementary.pdf]

Supplementary File

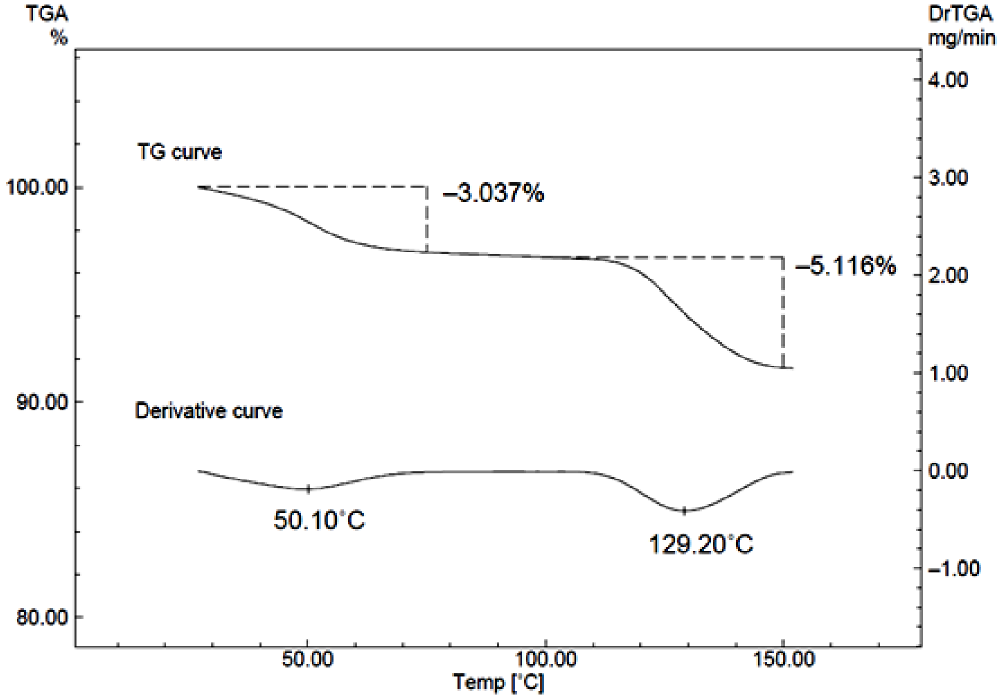

Figure S1. TG curves of *L. plantarum* EIR/IF–1 EPS fraction

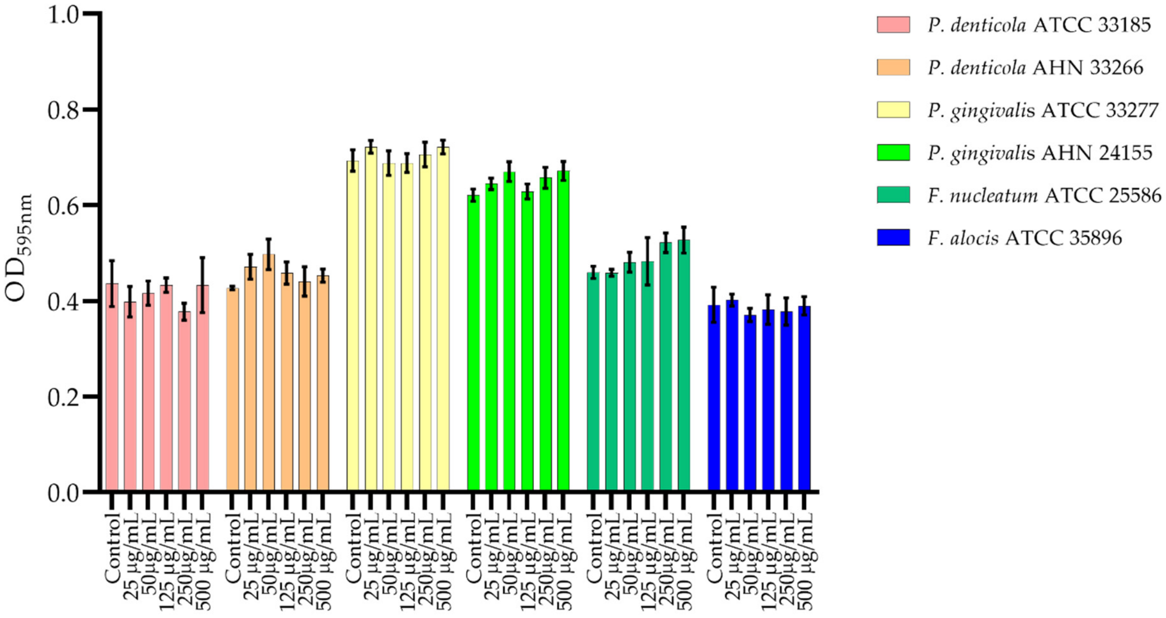

Figure S2. Testing of EPS fractions for growth of test strains. No antimicrobial activity was observed. Test strains were cultured in 96-well polystyrene plates for 48 h at 37 °C under anaerobic conditions with the indicated EPS concentrations in biofilm experiments.
